# Estrogen-containing contraceptive use and blood lead concentrations in a cohort of premenopausal individuals

**DOI:** 10.1016/j.envres.2025.122935

**Published:** 2025-09-25

**Authors:** Lauren E. Chapman, Mandy S. Hall, Arianna Foster, Donna D. Baird, Quaker E. Harmon, Robert O. Wright, Julio A. Landero, Renee Heffron, Lauren A. Wise, Ganesa Wegienka, Ruth J. Geller, Amelia K. Wesselink, Samantha Schildroth, Janet E. Hall, Erik J. Tokar, Kristen Upson

**Affiliations:** a College of Human Medicine, Michigan State University, East Lansing, MI, USA; b Department of Epidemiology and Biostatistics, College of Human Medicine, Michigan State University, East Lansing, MI, USA; c Epidemiology Branch, National Institute of Environmental Health Sciences, Research Triangle Park, NC, USA; d Department of Environmental Medicine, Icahn School of Medicine at Mount Sinai, New York, NY, USA; e Department of Medicine, University of Alabama at Birmingham, Birmingham, AL, USA; f Department of Epidemiology, Boston University School of Public Health, Boston, MA, USA; g Department of Public Health Sciences, Henry Ford Health, Detroit, MI, USA; h Clinical Research Branch, National Institute of Environmental Health Sciences, Research Triangle Park, NC, USA; i Mechanistic Toxicology Branch, Division of the National Toxicology Program, National Institute of Environmental Health Sciences, Research Triangle Park, NC, USA

**Keywords:** lead, Oral contraceptives, Bone health, Epidemiology, Cross-sectional study, Premenopausal women

## Abstract

After exposure, toxic metal lead is stored in the skeleton and is mobilized to systemic circulation with bone turnover. Given the bone-conserving properties of estrogen, we investigated whether current use of estrogen-containing contraception is associated with lower blood lead concentrations. We conducted a cross-sectional analysis using enrollment data from the Study of Environment, Lifestyle & Fibroids (SELF), a cohort of 1693 Black women ages 23–35 years enrolled in years 2010–2012. The study population was restricted to non-users of injectable hormonal contraception with questionnaire data on hormonal contraceptive use and laboratory data on whole blood lead concentrations (n = 1549). The geometric mean blood lead concentrations for current users of estrogen-containing contraception and non-users were 0.41 μg/dl (95 % CI: 0.39–0.43) and 0.51 μg/dl (95 % CI: 0.50–0.52), respectively. After adjusting for age, education, current smoking status, alcohol consumption, recency of injectable contraceptive hormone use, and recent birth using a multivariable linear regression model to estimate the percent difference in blood lead concentrations, current use of estrogen-containing contraception was associated with an 11 % lower blood-lead concentrations (95 % CI: −16 %, −5 %). In exploratory analyses considering contraceptive type, current combined oral contraceptive users (n = 187) had 10 % lower blood lead concentrations (95 % CI: −16 %, −4 %) and contraceptive vaginal ring/transdermal patch users (n = 33) had 18 % lower blood lead concentrations (95 % CI: −29 %, −5 %) compared with non-users. Given the known toxic effects of lead and the common use of estrogen-containing contraception, further research is warranted to confirm our observation of lower blood lead concentrations with current use of estrogen-containing contraception.

## 1. Introduction

Exposure to lead continues to be a public health concern (WHO, 2020). Lead is a well-documented toxicant; there is no safe level of exposure, and lead can affect all major organ systems (ATSDR, 2020). Lead can have far-reaching health effects because it mimics and interferes with calcium ions in critical cellular processes, including signal transduction, enzyme activation, neurotransmitter release, muscle contraction, and bone metabolism (ATSDR, 2020; Rocha and Trujillo, 2019). Through dysregulation of these molecular processes, lead exposure has been associated with increased risks of a range of conditions, including hypertension, cardiovascular disease, renal damage, decrements in cognition, behavioral problems, Parkinson’s disease, and schizophrenia in adults (ATSDR, 2020; Lamas et al., 2023; Lanphear et al., 2024). The general population is ubiquitously exposed to lead from contaminated air, dust, food, and water (ATSDR, 2020). After exposure, lead systemically circulates in red blood cells and accumulates in bone (Barry, 1975). However, lead does not remain sequestered in bone. Rather, during bone turnover, lead is mobilized back into systemic circulation, with bone lead contributing to 45 %–70 % of lead in blood (Gulson et al., 1995). Thus, physiologic states and medications associated with increased bone turnover, such as pregnancy (Gulson et al., 2003; Hertz-Picciotto et al., 2000; Moura and Goncalves Valente, 2002; Rothenberg et al., 1994; Schell et al., 2000), the postnatal period (Gulson et al., 1998), lactation (Gulson et al., 2003; Tellez-Rojo et al., 2002), menopause (Berkowitz et al., 2004; Hernandez-Avila et al., 2000; Nash et al., 2004; Silbergeld et al., 1988; Symanski and Hertz-Picciotto, 1995), and the injectable hormonal contraceptive depot medroxyprogesterone acetate (DMPA) (Upson et al., 2020) have been linked with increased blood lead concentrations.

In contrast, estrogen in hormonal contraception, such as combined oral contraception, contraceptive vaginal ring, and contraceptive transdermal patch, can exert bone-sparing effects in premenopausal women, reducing bone turnover (Herrmann and Seibel, 2010; Nappi et al., 2012). Estrogen inhibits bone resorption by suppressing both osteoclast formation and enhancing osteoblast activity that facilitates the synthesis of critical bone-building proteins, such as collagen, and the mineralization of newly formed bone (Kameda et al., 1997; Khosla et al., 2012). Given the bone-preserving effects of estrogen that could minimize the mobilization of lead from bone to blood, we hypothesized that current use of estrogen-containing contraception would be associated with lower blood lead concentrations compared with non-use. To our knowledge, only two studies have investigated estrogen-containing contraceptive use and blood lead concentrations, and these studies yielded mixed results (Akinloye et al., 2011; Iglesias et al., 2008). The discrepant results were likely due to small sample size and approach to statistical analyses as well as differences in patient age and biologic matrix used for lead measurement across studies. Hence, the purpose of the present study was to investigate the association between current estrogen-containing contraceptive use and blood lead concentrations using data from the Study of Environment, Lifestyle & Fibroids (SELF).

## 2. Material and methods

### 2.1. Study data and design

We conducted a cross-sectional analysis of current estrogen-containing contraceptive use and blood lead concentrations using enrollment data from SELF. SELF is an ongoing prospective study of 1693 women that was designed to investigate risk factors for the incidence and growth of uterine fibroids (Baird et al., 2015). The primary inclusion criteria for SELF were self-identifying as Black or African American, age 23–35 years, having an intact uterus, having no prior clinical diagnosis of fibroids, and being able to attend study clinic visits in Detroit, Michigan. We excluded those reporting any prior diagnoses of cancer requiring radiation or chemotherapy and those with lupus, Grave’s disease, Sjogren’s, scleroderma, or multiple sclerosis requiring medication. SELF was conducted in collaboration with Henry Ford Health in Detroit, Michigan, and a broad strategy was used to recruit participants from 2010 to 2012. The recruitment strategies included use of a study website, brochures, media advertisements, community events, and letters sent to those previously seen at Henry Ford Health. The Institutional Review Boards at the National Institute of Environmental Health Sciences, Henry Ford Health, and Boston University Medical Center approved the conduct of SELF and the collection of data for the present analyses; all SELF participants provided informed consent. As the SELF data were de-identified at Michigan State University, the conduct of research for the present study was deemed not to involve human subjects by the Human Research Protections Program (institutional review board).

At enrollment, participants attended a clinic visit during which trained study personnel measured height and weight and collected biological samples (Baird et al., 2015). Blood samples were subsequently analyzed for hemoglobin (g/dL) and serum 25-hydroxyvitamin D (25 (OH)D). Participants completed computer-assisted telephone and web-based questionnaires that ascertained information on demographic, lifestyle, early-life, and dietary factors, as well as contraceptive, pregnancy, and medical histories. All questionnaires were completed before or during the enrollment visit.

### 2.2. Estrogen-containing contraceptive use

During the computer-assisted telephone interview, each participant was asked about their contraceptive history, including a series of questions on the ever use of individual hormonal contraceptive methods that contain estrogen, specifically combined oral contraception (COC), contraceptive vaginal ring, and contraceptive transdermal patch. If the respondent reported yes to any of these methods, they were additionally asked if they were currently using the method. For oral contraception, participants were also asked the name of the birth control pill. We instructed participants to bring all prescription medications used in the past 24 h to the enrollment clinic visit. We recorded the prescription medications on the 24-h questionnaire and coded them using the Slone Drug Dictionary (Slone Epidemiology Center, Boston University, Boston, MA). We used these data in concert to determine current use of estrogen-containing contraception at the enrollment visit, and for oral contraception, the brand and dose of ethinyl estradiol (EE).

Using this information, we created a composite variable of current estrogen-containing contraceptive use (no, yes); the comparison group of non-users included both past estrogen-containing contraceptive users and never users. For use in exploratory analyses, we categorized the type of estrogen-containing contraception (non-user, COC, contraceptive vaginal ring/transdermal patch) as the pharmacokinetics for COCs differ from the contraceptive vaginal ring and transdermal patch. COCs are taken daily with hormone concentrations peaking 1.5–2 h after administration (Hampson, 2023). A contraceptive vaginal ring is retained vaginally for 21 days after insertion and allows for a low, steady, continuous dosing of estrogen (Kerns and Darney, 2011; U.S. Food & Drug Administration). A single contraceptive transdermal patch is dermally applied weekly for 3 weeks and delivers a more stable estrogen dose compared with COCs (Abrams et al., 2002; U.S. Food & Drug Administration). We also categorized the EE dose in COCs (non-user, 10–25 mcg, 30 mcg, and 35 mcg); current users of vaginal contraceptive ring and contraceptive transdermal patch were excluded when determining EE dose.

### 2.3. Blood lead concentrations

At the SELF enrollment study visit, non-fasting whole blood samples from 1664 study participants were collected (98 % of the original SELF cohort of 1693 participants). The sample collection protocol was designed to limit metal contamination and promote specimen stability (Upson et al., 2020). After collection, samples were stored at −20 °C at the National Institute of Environmental Health Sciences Repository, Experimental Pathology Laboratories, Inc. After a single thaw, they were shipped on dry ice to the Trace Metals Group at the Senator Frank R. Lautenberg Environmental Health Sciences Laboratory in the Department of Environmental Medicine and Public Health at the Icahn School of Medicine at Mount Sinai. Lead was quantified by inductively coupled plasma mass spectrometry using Agilent 8900-QQQ triple quadrupole instrument (Santa Clara, CA) (Geller et al., 2023). The daily limit of detection was calculated by the laboratory using the blood lead concentration at three times the standard deviation in the method blank. Lead was quantified above the detection limit in all samples. The daily limits of detection ranged from 0.001 to 0.003 μg/dL and the average intra-day coefficient of variation for lead was low (3.1 %) (Geller et al., 2023).

### 2.4. Study participants

Starting with the 1664 participants for whom whole blood was available for the measurement of lead, we excluded participants currently using DMPA at enrollment (n = 109) given our previous finding of higher blood lead levels among current DMPA users compared with non-users in SELF (Upson et al., 2020). We also excluded participants using non-contraceptive hormones (n = 2) and multiple types of contraceptive and non-contraceptive hormonal medications containing estrogen (n = 4). A total of 1549 participants were included in the present analyses.

### 2.5. Statistical analysis

We descriptively compared participant characteristics at enrollment between current estrogen-containing contraceptive users and nonusers. Among nonusers, we estimated the geometric mean (GM) blood lead concentrations and 95 % confidence intervals (CIs) by categories of participant characteristics; given the contribution of age and cigarette smoking to blood lead concentrations, the GMs were adjusted for these factors using multivariable linear regression.

We natural log-transformed the whole blood lead concentrations and conducted multivariable linear regression to estimate the percent difference in blood lead concentrations and 95 % CIs, comparing current estrogen-containing contraceptive users with nonusers. To express the estimates and 95 % CIs as percent differences, we used formula 100* [exp(β)-1]. We selected covariates for adjustment *a priori* based on associations reported in the literature on factors associated with estrogen-containing contraceptive use and blood lead concentrations (Geller et al., 2023; Lin and Lee, 2015). *A priori* adjustment factors were participant age (continuous), education (≤ high school or general education diploma (GED), some college or Associate’s or technical degree, ≥ Bachelor’s degree), current smoking status (no, yes), alcohol consumption in the past year (none, moderate, heavy), recency of past DMPA use (never, ≥3 years ago, <3 years ago), and birth in last year (no, yes) (Model 1). We additionally adjusted for factors associated with bone health (U.S. Department of Health and Human Services, 2004), including current vitamin D status using log_2_-transformed annual mean 25(OH)D concentrations (estimated using a cosinor model to account for seasonal changes) (Harmon et al., 2016), estimated total calcium intake from diet and supplements (<800, ≥800 mg/day), and a composite variable (no, yes) for history of medical conditions or current medication use associated with bone loss, specifically thyroid condition, anorexia nervosa, irritable bowel syndrome, anticonvulsant, thyroid hormone replacement, heparin, and glucocorticoid medication use (Model 2). All analyses were conducted using Stata version 15.1 (StataCorp, College Station, TX).

To evaluate the robustness of our results, we conducted several sensitivity analyses. First, we repeated the analyses restricting the study population to never-smokers (n = 1139) as another approach to account for confounding from cigarette smoking, which substantially contributes to blood lead concentrations (ATSDR, 2020). Second, we restricted the study population to those who have ever used estrogen-containing contraception (n = 1018). By comparing current users with past users (two or more years in the past), we sought to address the potential for confounding from unmeasured risk factors for lead exposure that vary between estrogen-containing contraceptive users and non-users. Third, we repeated the main analyses adjusting for hemoglobin to account for anemia status that may differ between current estrogen-containing contraceptive users and non-users. In blood, lead is stored in red blood cells (ATSDR, 2020) and current estrogen-containing contraceptive use is associated with reduced menstrual blood loss and decreased anemia risk (Bahamondes et al., 2015; Misunas et al., 2024). Fourth, we restricted the study population to those without contraindications for estrogen-containing contraceptive use (Centers for Disease Control and Prevention, 2010) (n = 1516). The contraindications available in SELF were a history of blood clot, angina, heart attack, or stroke. These contraindications are also associated with blood lead concentrations (Lamas et al., 2023). Fifth, we restricted the study population to those 25 years and older (n = 1322) as those younger than 25 years may be still acquiring peak bone mass (Heaney et al., 2000). Lastly, we restricted the study population to those without a history of conditions or medications associated with bone loss (n = 1415). We additionally explored the type of current estrogen-containing contraception as well as the EE dose of COCs in relation to blood lead concentrations.

## 3. Results

In the study sample, 14 % of SELF participants (n = 220) reported currently using estrogen-containing contraception at enrollment. Current estrogen-containing contraceptive users tended to have higher educational attainment (≥Bachelor’s degree), higher annual household income (>$50,000), to engage in moderate alcohol consumption, and to have never smoked compared with non-users (Table 1). Participants currently using estrogen-containing contraception, compared with non-users, also tended to have higher median 25(OH)D concentrations as well as lower frequency of anemia (hemoglobin <12 g/dl) and ever use of DMPA in the past.

The geometric mean blood lead concentration among all SELF participants at enrollment was 0.49 μg/dL (95 % CI: 0.48, 0.51 μg/dL). Among participants who did not use estrogen-containing contraception (n = 1329), those with lower education and lower household income as well as those who currently smoke and consumed heavy amounts of alcohol within the last year had higher geometric mean blood lead concentrations (Table 1). In addition, higher blood lead concentrations at the SELF enrollment visit were observed among those who had given birth in the past year and among those with high blood lead levels detected during infancy or childhood.

The geometric mean blood lead concentration was lower for current estrogen-containing contraceptive users (0.41 μg/dL, 95 % CI: 0.39–0.43) than non-users (0.51 μg/dL, 95 % CI: 0.50–0.52) (Table 2). After multivariable adjustment, current estrogen-containing contraceptive users had blood lead concentrations that were 11 % lower compared with nonusers (95 % CI: −16 %, −5 %) (Model 1). After further adjustment for 25(OH)D concentration, dietary calcium intake, and medical conditions and medications associated with bone health (Model 2), current use of estrogen-containing contraception was associated with 10 % lower blood lead concentrations (95 % CI: −16 %, −4 %).

In our sensitivity analyses, we observed a similar percent difference in blood lead concentrations to that obtained in the main analyses comparing current estrogen-containing contraceptive users and non-users after: (i) restricting the study population to never smokers (10 % lower, 95 % CI: −15 %, −4 %); (ii) comparing current estrogen-containing contraceptive users with past estrogen-containing contraceptive users who had discontinued ≥2 years prior to enrollment (12 % lower, 95 % CI: −17 %, −6 %); (iii) additionally adjusting for hemoglobin (11 % lower, 95 % CI: −17 %, −6 %); (iv) excluding those with a contraindication for estrogen-containing contraceptive use (11 % lower, 95 % CI: −16 %, −5 %); (v) restricting study participants to those 25 years and older (12 % lower, 95 % CI: −18 %, −6 %); and (vi) including only those without a history of conditions or medications associated with bone loss (11 % lower, 95 % CI: −17 %, −5 %) (Table 2).

In our exploratory analysis of the type of estrogen-containing contraception, blood lead concentrations were 10 % lower (95 % CI: −16 %, −4 %) among current users of COCs (n = 187) and 18 % lower (95 % CI: −29 %, −5 %) among current users of contraceptive vaginal ring or transdermal patch (n = 33) compared with non-users of estrogen-containing contraception (Table 3). When we explored the COC EE dose, the percent difference in blood lead concentrations was generally similar in magnitude across EE doses. For example, compared to non-users, those using COCs containing 10–25 mcg EE had 13 % lower lead concentrations (95 % CI: −22 %, −3 %), and those using COCs containing 35 mcg EE had 12 % lower lead concentrations (95 % CI: −22 %, −0 %) (Table 4).

## 4. Discussion

In the present study, we observed that current use of estrogen-containing contraception was associated with lower blood lead concentrations. This association may be explained by the known bone-preserving effects of estrogen, and bone being an endogenous reservoir for circulating lead. After exposure, lead replaces calcium in the primary crystalline matrix and is stored in bone (ATSDR, 2020). However, bone remodeling is a continuous, dynamic process, with bone resorption (breakdown) and reformation (Nappi et al., 2012; U.S. Department of Health and Human Services, 2004). Lead follows the physiology of calcium storage and release in bone, and lead is released from bone to systemic circulation with bone resorption (Silbergeld, 1991; Silbergeld et al., 1993). Endogenous estrogen, on the other hand, inhibits bone resorption and maintains bone formation (Kameda et al., 1997; Khosla et al., 2012) and could reduce the mobilization of lead stored in bone (Silbergeld, 1991; Silbergeld et al., 1993). Since current estrogen-containing contraceptive use has been generally associated with decreases in bone remodeling as assessed by bone turnover markers (Herrmann and Seibel, 2010), it is plausible that estrogen-containing contraception could also reduce skeletal mobilization of lead to systemic circulation. While we did not have data on bone turnover markers in SELF, in our exploratory analysis, we did observe a stronger association (18 % lower blood lead concentrations) with current use of the 21-day contraceptive vaginal ring or weekly-applied transdermal patch, which provide a more stable concentration of estrogen, compared with the association for COC use (10 % lower blood lead concentrations), which exhibits daily estrogen peaks and troughs (Abrams et al., 2002; Kerns and Darney, 2011).

To our knowledge, only two cross-sectional studies have previously evaluated estrogen-containing contraceptive use and blood lead concentrations (Akinloye et al., 2011; Iglesias et al., 2008). The first study was conducted among 174 adolescent clinic patients in New York City, ages 13–21 years (Iglesias et al., 2008). That study reported a slightly lower mean lead concentration among oral contraceptive pill users (n = 25, 1.2 μg/dl) compared with those not using hormonal contraception (n = 121, 1.5 μg/dl). However, the small study size, lack of adjustment for confounding, and study population of adolescent patients attaining peak bone mass with high baseline bone turnover (Herrmann and Seibel, 2010) may have decreased the ability of that study to detect a difference in blood lead concentrations. The other study was conducted in Nigeria among 150 family planning patients ages 18–40 years and compared users of oral contraception (n = 50), injectable hormonal contraception (n = 25), and intrauterine device (n = 25) to those not using contraception (n = 50) (Akinloye et al., 2011). Across the contraceptive groups, the authors reported similar mean serum lead concentrations. However, the interpretation of results from that study are difficult given the lack of statistical adjustment for confounding, the comparison of mean concentrations for lead concentrations that are typically right-skewed, and the potential for measurement error with the measurement of lead in serum; as > 98 % of lead in blood resides in red blood cells (Hu et al., 1998), measurement error can be introduced from low serum lead concentrations near the detection limit and hemolysis (ATSDR, 2020). Thus, in concert, these aspects of study design may explain the discrepant results across studies. Our observation of lower blood lead concentrations with current estrogen-containing contraceptive use is consistent with epidemiologic studies reporting an association in the opposite direction – higher blood lead concentrations – with hypoestrogenic physiologic states, including lactation (Gulson et al., 2003; Tellez-Rojo et al., 2002) and menopause (Berkowitz et al., 2004; Hernandez-Avila et al., 2000; Nash et al., 2004; Silbergeld et al., 1988; Symanski and Hertz-Picciotto, 1995), as well as medications that reduce ovarian estrogen production such as current injectable hormonal contraceptive DMPA use (Upson et al., 2020).

Our study had limitations. First, given the cross-sectional study design, we could not establish the temporal ordering for estrogen-containing contraceptive use and blood lead levels. However, there is little biological rationale for reverse causation (i.e., blood lead leading to estrogen-containing contraceptive use) and lead measured in whole blood characterizes lead exposure over the past month (Graziano, 1994; Todd et al., 1996). Second, the observation of lower blood lead concentrations among current estrogen-containing contraceptive users may be explained by these participants having less exposure to lead than non-users. For example, in SELF, some predictors of higher blood lead concentrations (e.g., lower education attainment, current smoking) were less prevalent among estrogen-containing contraceptive users compared with non-users. That said, in our sensitivity analyses comparing current and past estrogen-containing contraceptive users, the observation of lower blood lead concentrations with current estrogen-containing contraceptive use persisted, providing reassurance. Third, multivariable adjustment for cigarette smoking may not completely account for confounding by this factor that is a substantial contributor to blood lead levels (ATSDR, 2020). This concern is minimized by the similar association observed in a sensitivity analysis restricting the study population to never smokers to that of the main analyses. Fourth, in SELF, information on duration of current estrogen-containing contraceptive use was not available. This information would have contributed to understanding the duration of estrogen-containing contraceptive use needed after initiation to contribute to lower bone turnover and less skeletal lead mobilization. Lastly, our study did not have data on markers of bone turnover that would have provided valuable information to support the hypothesized mechanism.

Our study had several notable strengths. The present analyses were conducted among a large cohort of Black premenopausal participants with a distribution of blood lead concentrations similar to those reported in the National Health and Nutrition Examination Survey for US Black women aged 23–34 years in 2011–2012 (Upson et al., 2020). Thus, the results from the present analyses may be generalizable to individuals beyond those residing in the Detroit, Michigan, area. In addition, the study population comprised participants aged 23–35 years at enrollment, 78 % of whom were older than the average age of peak bone acquisition (approximately age 25 years) (Heaney et al., 2000). This age range allowed for the detection of an association between estrogen-containing contraceptive use and lower blood lead concentrations in a population no longer susceptible to pubertal effects on bone turnover rates. Furthermore, the present study benefitted from the extensive, detailed data collected in SELF on contraceptive use and relevant covariate data, including detailed data on COC EE dose. Lastly, the concentrations of lead in whole blood were measured by a laboratory with extensive experience in the measurement of toxic metals and is one of the NIH Human Health Exposure Analysis Resources (HHEAR) labs.

## 5. Conclusion

In a large cohort of premenopausal participants, current estrogen-containing contraceptive use was associated with lower blood lead concentrations. The consistent results across several sensitivity analyses support the validity of the finding. Given the widely recognized anti-resorptive effects of estrogen on bone and promotion of bone formation, our results suggest that use of estrogen-containing contraception may limit the skeletal release of lead into systemic circulation. These results are intriguing given the known toxic effects of lead and epidemiologic studies reporting an association in the opposite direction – higher blood lead concentrations – with physiologic states and medications unique to reproductive-age individuals that induce increased bone turnover, such as pregnancy, lactation, menopause, and use of the injectable hormonal contraceptive DMPA. However, further research is warranted to understand the potential clinical implications of our findings. This includes confirming our results, verifying the mechanism underlying our observations, and determining whether lower blood lead concentrations are observed with the use of other estrogen-containing medications such as those used to manage menopausal symptoms.

## Figures and Tables

**Table 1:** Participant characteristics at enrollment by current estrogen-containing contraceptive use, Study of Environment, Lifestyle & Fibroids (SELF), 2010–2012 (n = 1549).

	Current estrogen-containing contraceptive use^a^		Blood lead (μg/dl) among those not currently using estrogen-containing contraception (n = 1329) Adjusted GM (95 % CI)^b^
Characteristic	Yes n = 220 n (%)	No n = 1329 n (%)	
Age at enrollment visit (years)			
23-25	56 (25)	282 (21)	0.48 (0.46, 0.51)^c^
26-28	60 (27)	327 (25)	0.52 (0.50, 0.55)^c^
29-31	52 (24)	368 (28)	0.49 (0.47, 0.52)^c^
32-35	52 (24)	352 (26)	0.54 (0.52, 0.57)^c^
Education			
≤HS or GED	18 (8)	311 (23)	0.58 (0.56, 0.61)
Some college or Associate/technical degree	95 (43)	678 (51)	0.51 (0.49, 0.52)
Bachelor’s, Master’s or doctoral degree	107 (49)	340 (26)	0.46 (0.44, 0.48)
Total annual household income (US$)			
<20,000	56 (25)	636 (48)	0.55 (0.53, 0.57)
20–50,000	101 (46)	480 (36)	0.49 (0.47, 0.51)
>50,000	63 (29)	213 (16)	0.46 (0.43, 0.48)
Smoking status			
Never	199 (90)	940 (71)	0.45 (0.44, 0.46)^d^
Former	9 (4)	109(8)	0.53 (0.48, 0.57)^d^
Current, <10 cigarettes/day	10 (5)	204 (15)	0.75 (0.71, 0.80)^d^
Current, ≥10 cigarettes/day	2 (1)	76 (6)	0.84 (0.76, 0.93)^d^
Alcohol consumption last year^e^			
None	39 (18)	408 (31)	0.48 (0.46, 0.51)
Moderate	146 (66)	649 (49)	0.51 (0.49, 0.52)
Heavy	35 (16)	272 (20)	0.56 (0.54, 0.60)
Body mass index (kg/m^2^)^f^			
<25.0	51 (23)	260 (20)	0.52 (0.49, 0.55)
25.0 to <30.0	57 (26)	274 (21)	0.52 (0.50, 0.55)
30.0 to <35.0	61 (28)	269 (20)	0.52 (0.49, 0.54)
35.0 to <40.0	26 (12)	229 (17)	0.49 (0.46, 0.52)
≥40.0	25 (11)	294 (22)	0.50 (0.47, 0.52)
Exercise in past 12 months^f^			
Low	31 (14)	219 (17)	0.50 (0.47, 0.53)
Low to moderate	58 (26)	299 (23)	0.51 (0.48, 0.53)
Moderate	51 (23)	351 (26)	0.53 (0.51, 0.56)
High	50 (23)	250 (19)	0.49 (0.47, 0.52)
Very high	29 (13)	207 (16)	0.52 (0.49, 0.55)
Daily total calcium intake (mg/day) during prior year^g^			
Low (<800 mg/day)	118 (54)	699 (53)	0.51 (0.49, 0.53)
High (≥800 mg/day)	102 (46)	630 (47)	0.51 (0.50, 0.53)
Estimated annual mean 25(OH)D concentration^f^			
<Median (15.25 ng/ml)	76 (35)	693 (52)	0.52 (0.50, 0.54)
≥Median (15.25 ng/ml)	143 (65)	632 (48)	0.50 (0.48, 0.52)
Birth in past year			
No	208 (95)	1254 (94)	0.51 (0.49, 0.52)
Yes	12 (5)	75 (6)	0.59 (0.54, 0.65)
Conditions or medication use associated with bone loss^h^			
No	199 (90)	1216 (92)	0.51 (0.50, 0.53)
Yes	21 (10)	113 (9)	0.48 (0.45, 0.52)
Anemia (hemoglobin <12 g/dl)^f^			
No	169 (79)	906 (69)	0.52 (0.50, 0.53)
Yes	46 (21)	404 (31)	0.49 (0.47, 0.51)
Told by doctor had high lead levels when infant or child^f^			
No	211 (100)	1228 (98)	0.50 (0.49, 0.52)
Yes	0 (0)	31 (2)	0.61 (0.52, 0.71)
Recency of past DMPA use^f^			
Never use	160 (73)	791 (60)	0.50 (0.48, 0.51)
<3 years ago	17 (8)	104 (8)	0.55 (0.50, 0.60)
≥3 years ago	43 (19)	433 (33)	0.53 (0.50, 0.55)

Abbreviations: CI, confidence interval; DMPA, depot medroxyprogesterone acetate, GED, general equivalency diploma; GM, geometric mean; HS, high school; 25(OH)D, 25-hydroxyvitamin D.

a Use of combined oral contraception, contraceptive vaginal ring or patch at enrollment visit.

b Geometric mean blood lead concentration adjusted for age (continuous) and current smoking (no, yes).

c Geometric mean blood lead concentration adjusted only for current smoking (no, yes).

d Geometric mean blood lead concentration adjusted only for age (continuous).

e Moderate alcohol intake defined as more than none but less than heavy alcohol consumption (≥6 drinks per day when drinking alcohol or ≥4 drinks at a single sitting twice per month or more).

f Missing data on body mass index (n = 0 exposed, n = 3 unexposed), exercise in past 12 months (n = 1 exposed, n = 3 unexposed); estimated annual mean 25(OH)D concentration (n = 1 exposed, n = 4 unexposed); hemoglobin (n = 5 exposed, n = 19 unexposed); told by doctor had high blood lead levels when infant or child (n = 9 exposed, n = 70 unexposed); recency of past DMPA use (n = 0 exposed, n = 1 unexposed).

g Total calcium intake includes intake from both dietary and supplement sources.

h Self-reported history of medical conditions (thyroid condition, anorexia, irritable bowel syndrome) or current medication use (anticonvulsant, thyroid hormone replacement, heparin, or glucocorticoid) associated with bone loss.

**Table 2:** Current estrogen-containing contraceptive use and blood lead concentrations in the Study of Environment, Lifestyle & Fibroids (SELF), 2010–2012.

Analyses	Current estrogen-containing contraceptive use	n (%)	Blood lead (μg/dl) GM (95 % CI)	Model 1^a^ % Difference (95 % CI)	Model 2^b^ % Difference (95 % CI)
Main analysis (n = 1549)	No	1329 (86)	0.51 (0.50–0.52)	(Reference)	(Reference)
	Yes	220 (14)	0.41 (0.39–0.43)	−11 (−16, −5)	−10 (−16, −4)
Restricted to never smokers (n = 1139)^c^	No	940 (83)	0.45 (0.44–0.46)	(Reference)	(Reference)
	Yes	199 (17)	0.40 (0.38–0.42)	−10 (−15, −4)	−9 (−15, −3)
Restricted to ever users of estrogen-containing contraception (n = 1018)^d^	No	798 (78)	0.50 (0.49–0.52)	(Reference)	(Reference)
	Yes	220 (22)	0.41 (0.39–0.43)	−12 (−17, −6)	−11 (−17, −4)
Additional hemoglobin adjustment (n = 1525)^e^	No	1310 (86)	0.51 (0.50–0.52)	(Reference)	(Reference)
	Yes	215 (14)	0.41 (0.39–0.43)	−11 (−17, −6)	−11 (−16, −5)
Among participants without contraindications for estrogen-containing contraceptive use^f^ (n = 1516)	No	1296 (85)	0.51 (0.50, 0.52)	(Reference)	(Reference)
	Yes	220 (15)	0.41 (0.39, 0.43)	−11 (−16, −5)	−10 (−15, −4)
Among participants ages ≥25 years (n = 1322)	No	1135 (86)	0.52 (0.50, 0.53)	(Reference)	(Reference)
	Yes	187 (14)	0.41 (0.39, 0.43)	−12 (−18, −6)	−11 (−17, −5)
Among participants without a history of conditions or medications associated with bone loss (n = 1415)^g^	No	1216 (86)	0.51 (0.50, 0.53)	(Reference)	(Reference)
	Yes	199 (14)	0.41 (0.39, 0.43)	−11 (−17, −5)	−10 (−16, −4)

Abbreviations: GM, geometric mean; CI, confidence interval.

a Adjusted for age (continuous), education (≤HS/GED, some college/Associate’s degree, Bachelor’s/Master’s/Doctoral degree), current smoking (no, yes), alcohol use in last year (none, moderate, heavy), recency of past depot medroxyprogesterone acetate use (never, ≥3 years ago, <3 years ago), and birth in last year (no, yes).

b Adjusted for age (continuous), education (≤HS/GED, some college/Associate’s degree, Bachelor’s/Master’s/Doctoral degree), current smoking (no, yes), alcohol use in last year (none, moderate, heavy), recency of past depot medroxyprogesterone acetate use (never, ≥3 years ago, <3 years ago), birth in last year (no, yes), log_2_-transformed estimated annual mean 25(OH)D concentrations (continuous), total calcium intake (<800, ≥800 mg/d), and self-reported history of medical conditions (thyroid condition, anorexia, irritable bowel syndrome) or current medication use (anticonvulsant, thyroid hormone replacement, heparin, or glucocorticoid) associated with bone loss (no, yes).

c Additionally adjusted for passive smoking exposure defined as hours per week the participant smelled tobacco smoke (0, 1, 2–6, ≥7 h).

d Current or past use (two or more years in the past) of combined oral contraceptives, contraceptive vaginal ring, or contraceptive transdermal patch.

e Hemoglobin measurement missing for 24 participants.

f Contraindications for estrogen-containing contraception include history of blood clot, angina, heart attack, or stroke.

g Self-reported history of medical conditions (thyroid condition, anorexia, irritable bowel syndrome) or current medication use (anticonvulsant, thyroid hormone replacement, heparin, or glucocorticoid) associated with bone loss.

**Table 3:** Type of estrogen-containing contraceptive use and blood lead concentrations in the Study of Environment, Lifestyle & Fibroids (SELF), 2010–2012.

	n (%)	Blood lead (μg/dl) GM (95 % CI)	Model 1^a^ % Difference (95 % CI)	Model 2^b^ % Difference (95 % CI)
Type of estrogen-containing contraception				
Non-user	1329 (86)	0.51 (0.50–0.52)	(Reference)	(Reference)
Current COC use	187 (12)	0.42 (0.39–0.44)	−10 (−16, −4)	−9 (−15, −2)
Current contraceptive vaginal ring or transdermal patch use	33 (2)	0.37 (0.33–0.42)	−18 (−29, −5)	−17 (−28, −4)

Abbreviations: COC, combined oral contraception; GM, geometric mean; CI, confidence interval.

a Adjusted for age (continuous), education (≤HS/GED, some college/Associate’s degree, Bachelor’s/Master’s/Doctoral degree), current smoking (no, yes), alcohol use in last year (none, moderate, heavy), recency of past depot medroxyprogesterone acetate use (never, ≥3 years ago, <3 years ago), and birth in last year (no, yes).

b Adjusted for age (continuous), education (≤HS/GED, some college/Associate’s degree, Bachelor’s/Master’s/Doctoral degree), current smoking (no, yes), alcohol use in last year (none, moderate, heavy), recency of past depot medroxyprogesterone acetate use (never, ≥3 years ago, <3 years ago), birth in last year (no, yes), log_2_-transformed estimated annual mean 25(OH)D concentrations (continuous), total calcium intake (<800, ≥800 mg/d), and self-reported history of medical conditions (thyroid condition, anorexia, irritable bowel syndrome) or current medication use (anticonvulsant, thyroid hormone replacement, heparin, or glucocorticoid) associated with bone health (no, yes).

**Table 4:** Combined oral contraception ethinyl estradiol dose and blood lead concentrations in the Study of Environment, Lifestyle & Fibroids (SELF), 2010–2012.

	n (%)	Blood lead (μg/dl) GM (95 % CI)	Model 1^a^ % Difference (95 % CI)	Model 2^b^ % Difference (95 % CI)
Current COC ethinyl estradiol dose^c^				
0 mcg (non-user)	1329 (89)	0.51 (0.50–0.52)	(Reference)	(Reference)
10-25 mcg	62 (4)	0.40 (0.36–0.44)	−13 (−22, −3)	−12 (−21, −2)
30 mcg	56 (4)	0.42 (0.38–0.47)	−6 (−17, 5)	−5 (−15, 7)
35 mcg	50 (3)	0.41 (0.37–0.46)	−12 (−22, 0)	−11 (−21, 0)

Abbreviations: COC, combined oral contraception; GM, geometric mean; CI, confidence interval.

a Adjusted for age (continuous), education (≤HS/GED, some college/Associate’s degree, Bachelor’s/Master’s/Doctoral degree), current smoking (no, yes), alcohol use in last year (none, moderate, heavy), recency of past depot medroxyprogesterone acetate use (never, ≥3 years ago, <3 years ago), and birth in last year (no, yes).

b Adjusted for age (continuous), education (≤HS/GED, some college/Associate’s degree, Bachelor’s/Master’s/Doctoral degree), current smoking (no, yes), alcohol use in last year (none, moderate, heavy), recency of past depot medroxyprogesterone acetate use (never, ≥3 years ago, <3 years ago), birth in last year (no, yes), log_2_-transformed estimated annual mean 25(OH)D concentrations (continuous), total calcium intake (<800, ≥800 mg/d), and self-reported history of medical conditions (thyroid condition, anorexia, irritable bowel syndrome) or current medication use (anticonvulsant, thyroid hormone replacement, heparin, or glucocorticoid) associated with bone health (no, yes).

c Excluding current users of contraceptive vaginal ring or contraceptive transdermal patch.

## Data Availability

Participant data that underlie the primary results reported in this article can be requested for purposes of replication or meta-analysis by emailing co-authors baird@niehs.nih.gov and quaker.harmon@nih.gov. All data releases will require a data use proposal and a data use agreement and will comply with the IRB and consent of the SELF study, which may require omission of some data elements. The NIH IRB may be asked to review the request and study consent forms to approve a data transfer.
